# Barrel rifling node offset detection and subsequent optimization based on thin film in-mold decoration characteristics of the Johnson–Cook model

**DOI:** 10.1038/s41598-023-47467-0

**Published:** 2024-10-18

**Authors:** Hanjui Chang, Guangyi Zhang, Yue Sun, Shuzhou Lu

**Affiliations:** 1 Department of Mechanical Engineering, College of Engineering, Shantou University, Shantou, 515063 China; 2 Intelligent Manufacturing Key Laboratory of Ministry of Education, Shantou University, Shantou, 515063 China

**Keywords:** Applied mathematics, Scientific data, Statistics, Chemical engineering, Mechanical engineering

## Abstract

In this paper, a nodal detection method for the detection and optimization of barrel helix offsets is proposed. The barrel used in this experiment is a 6-helix barrel. Moreover, the special properties of the film of Polyetheretherketone (PEEK) material are used to cover the surface of the barrel helix with a virtual in-mold decoration (IMD) film, and the unique nature of the film die offset in the IMD is utilized to detect the position of the barrel helix. The area with a large die index is the area with a large helix offset in the barrel, and the IMD die index is introduced to quantify the data. The IMD die index is used to determine the helix offset of the damaged barrel. The novelty of this work is that each node can use the die index to efficiently detect the position of the barrel helix deviation, carry out subsequent optimization and repair work through the optimization of the injection molding parameters and the design optimization of the barrel and verify the experiment by comparing the results. Through the steady-state simulation research mode, different permutations and combinations of the two process parameters are simulated to study their effects. Quantitative reference indicators include but are not limited to dependent variables such as the fluid flow velocity, shear rate, temperature distribution and phase transition, and the shear heating process of the injection screw is taken into account in the mold flow analysis to ensure that the die index value is more accurate. It can be seen from the analysis results that the temperature of the barrel changes after the groove depth and groove width are changed, and the effect ratio of the groove depth is lower than that of the groove width in the same degree of size change.

## Introduction

The automatic rifle barrel is mainly composed of three parts: a rifling bullet, a lead sleeve and a steel core. Rifling is used to guide a number of spiral protrusions or grooves at a certain inclination angle to the axis of the body tube on the breech wall. The function of rifling is to make the bullet rotate when it is shot out of the muzzle. The function is to ensure the accuracy of the warhead. The cross-sectional profile of rifling commonly used on the inner breech cross-section is approximately a rectangle and is called rectangular rifling. The convex part of the spiral groove is called the white line, and it has a width. The groove under the concave is called the black line, the top arc is centered around the inner chamber cross-section, and the two sides of the black line are parallel to the radius of the midpoint of the black line. The purpose is to increase the strength of the bullet casing, and the width b of the black line is generally greater than the width a of the white line. The specific value ratio is b = (1.5 ~ 2.9)a, and the distance between the yin and yang lines in the radius direction is the rifling depth. To reduce the effect of the stress value at the end of the rifling, the junction of the yin and yang lines is usually connected with rounded corners, and the radius R of the fillet corners is 1/2 the rifling width. One side of the yang line, which is called the guide side of the rifling, gives the projectile a certain rotational force.

In the above discussion, the concept of rifling is introduced, and the size of the design and rifling processing is studied more than the rifling design. In metalworking analysis, the inclusion of body tubes, rifling, etc., beyond elastic bands can be regarded as rigid bodies, and when the bullet is squeezed into the rifling when moving in the barrel, contact with the rifling components is needed. The frictional contact of the bullet is studied. Since the friction coefficient between the bullet and the barrel bore is not constant, the bullet movement time is short, and the deformation speed is fast. When the bullet moves in the chamber, the time difference between the relative movement of the gun and the bullet is within 1.5 s, the relative movement speed is large, and the shooting speed is approximately 700 ~ 800 m/s. After shooting, the pressure in the barrel is 200 ~ 400 MPa. The rifling extrusion force promotes the interaction between the barrel and the bullet, the rifling cylinder and the barrel rifling squeeze each other, and the material undergoes high-temperature, high-speed transient deformation and high contact pressure friction, which makes experimental methods for rifling force changes and rifling shifts within the rifling more difficult to study. To solve the accurate detection of rifling displacement in machining, it is also necessary to calculate the machining stress for different types of rifling.

Different rifling types vary greatly in the pressure variation in the experiment and in the machining accuracy. According to the ratio of rifling depth, rifling can be divided into two types: shallow rifling and deep rifling. Shallow rifling indicates that the elastic belt has a low cutting resistance, and it is easier to wipe the inner cavity after shooting. The disadvantage of shallow rifling is that the area of the guide side is small, and the rifling easily wears, which affects the life of the body tube. It is generally believed that shallow rifling is suitable for automatic weapons with a low dry maximum bore pressure and muzzle velocity. The advantages and disadvantages of deep rifling are the opposite of those of shallow rifling. It is generally believed that deep rifling should be implemented in automatic weapons with high maximum bore pressures and muzzle velocities to meet the strength needs of rifling and cartridge belts. According to the change in the width of the rifling black line along the inner rifling axis, rifling can be divided into two types: equal width rifling and wedge rifling. In the current study, the rifling of most automatic weapons has a constant width and depth along the entire length of the rifling, and its processing technology is relatively good. Wedge rifling refers to the rifling in which the black section is wide and gradually narrows in the direction of the rifling, and the rifling depth gradually becomes shallower in the linear relationship from the back to the rifling direction, also known as the gradual rifling. The aim is to reliably seal the powder gas to help increase the muzzle velocity. The body tube of the type 56 14.5 anti-aircraft machine gun is divided into three sections: front, middle and rear. The width of the black line is narrower than that of the middle and rear sections, and the diameter of the black and white lines in the front section is also smaller than that of the middle and rear sections. In this way, when the bullet reaches the front tube, the cartridge belt can be compressed to reliably hold the air, which is conducive to increasing the muzzle velocity. American naval guns larger than 127 mm automatically had variable white lines and were later widened only on the white lines of equip-winding rifling. Generally, it is carried out according to the relationship of 2.032 mm widening every 25.4 m in the length direction. For example, the rectangular rifling in Table 1 represents Type 59 7.62 pistol and US M165.56 automatic. Through previous research, the disadvantage of wedge rifling is that it is extremely difficult to process, so its application is limited. Therefore, the bullet research process is limited to the influence of rectangular rifling and other types of rifling (such as trapezoidal rifling, polygonal arc rifling, etc.) on the high precision requirements of the process.

**Table 1:** Comparison table of the values of the three types of barrels.

Gun name	Type 59 7.62 pistol	US M165.56 automatic rifle	Type 56 14.5 anti-aircraft machine gun
Rifling rotation direction and number of strips	Right-4	Right-6	Right-8
Roundness	240 ± 1.3	305	420 ± 10
The diameter of the black line is D (mm)	7.92	13	14.93
Male wire diameter d (mm)	7.62	12.66	14.5
Rifling depth t (mm)	0.15	0.37	0.215

Mold-flow simulations and nodal simulations can be used to determine the positional changes in the experiments, such as temperature stress changes and offset changes during rifling. The simulations are implemented in COMSOL. It is a new idea to study the rifling extrusion offset deformation process by stress simulation software and Moldex3d mold flow simulation. Because finite element simulation can be conducted to solve the problem of instantaneous dynamic changes that cannot be observed by the test, such as the stress‒strain change of the rifling machining process, it is increasingly common to use finite line simulation software to study the problems of the rifling forming process. For example, by constructing a thermodynamic coupling finite element simulation model of rifling and body tubes in finite element simulation software, the change law of thermal coaction on rifling processing under metal cutting conditions is calculated and analyzed.

The above analysis of the optimization possibility of the machining accuracy of rectangular rifling and the measurement of the rifling data of different types of rifles can be performed. The purpose of this experiment is to improve the accuracy of rectangular rifling. The magnitude of the extrusion resistance of the initial movement of the rifling and the magnitude of the friction between the inner wall surfaces of the barrel are related to the material properties, the slope size and the form of the rifling. Moreover, the extrusion process includes a dynamic process. The rifling force time is short. Additionally, the force is large and has many forms, which makes the test of rifling offset particularly difficult. To analyze the experimental results and verify the theoretical simulation, the rifling offset measurement experiment is divided into three parts: a rifling forming optimization experiment, a nodal offset test experiment of the rifling, and an injection temperature optimization test of the rifling. The traditional test method is difficult to implement, and the relative cost is much greater. The use of the above three types of experiments for rifling tests, analysis and use under certain engineering conditions, combined with theoretical results, can greatly reduce the cost.

According to the above summary, the three categories can reduce the cost of the experimental method and the finite element and mold flow analysis performed by previous researchers. In this paper, the problem of squeezing in small-bore US M165.56 automatic 6 rifles is examined. From three perspectives, namely, theoretical analysis, numerical simulation and experimental verification, the interaction between the rifling and the barrel of a small-caliber rifling under different rifling cross-sectional shapes was analyzed. Effectively improving the machining offset of rectangular rifling through new processing methods and developing better polymer materials are important for understanding the mechanism of gun wire offsets during rifling processes. To verify the rifling offset position, the Johnson–Cook model was implemented in COMSOL software. This model was used to make assumptions regarding the stress distribution, simulate the offset model when rifling is shifted, and obtain the relevant offset barrel rifling node optimization die index diagram. Then, the results were compared with the die flow software results to verify the accuracy of the node technology.

## Literature review

In 2010, Chen et al.^1^ found that heat transfer in IMD molding was hindered by studying the molding of the cavity shapes. They concluded that the initial cavity temperature and shape affect the melt-film interface temperature when the thermal conductivity of the polycarbonate film changes by using the optimal matching of injection molding coolant, melt temperature and IMD film thickness. In 2010, Shuai and Haiyan et al.^2^ evaluated the properties of fiber lamellar composites and the ballistic properties after repair. This shows that reimplantation delamination during repair has less effect on the ballistic performance, which proves that the repaired ballistic performance of more fiberboard is better than that of baseline repair in design and subsequent repair. In 2011, Martinez et al.^3^ studied how the properties of textiles during pressure drop affect the filling stage of plastic materials. This shows that the fusion pressure drop of plastic melting and textiles is higher than that of conventional injection molding due to the stress of compressing foam material in addition to the compression of the plastic material. In 2020, Mahdi et al.^4^ studied the effects of the two patch adhesive systems on low temperature and room temperature curing and showed that the limited adhesion ability of the room temperature curing system limits the repair performance. Moreover, the strength provided by low transmission curing to the repair system allows the armor to be restored to the original structural properties. In 2018, Abtew et al.^5^ studied the effect of different injection parameters on the formability of multilayer composite materials and showed that the quilting of Mond-shaped materials provided very little tensile value. As the density increases to form harder layers to resist deformation, unstitched and quilted preforms have the highest and lowest rates of material deformation recovery, respectively. This shows that with the decrease in the length of the composite suture line, the deformation recovery rate of the material decreases.

In 2020, Majumdar^6^ investigated the influence of material and structural parameters on the use of new fibers. This includes surface treatment to increase friction in the yarn and the use of knitted fabrics to enhance structural integrity. This shows that the coil-free UD fabric structure can dissipate stress waves faster in terms of impact.

In 2020, Crouch^7^ studied the relationship between the fiber diameter length and ceramic density and monitored the performance between the adjacent layers of the backing plates by relevant quality control procedures, indicating the importance of the interlayer gap size of the fibers in the stacking system and the matching accuracy between the rigid plates and the molding process. In 2021, Li and Augusto^8^ proposed a model for estimating the number of bending cycles needed for lateral buckling and showed the correlation between the buckling and the number of bending cycles. Based on this model, the influence of the axial compressive properties and friction coefficient on the number of cycles and the stress during buckling are discussed. In 2021, Bhat, Naveen^9^ studied the materials of the armed system and found suitable material properties. Multi-layer fibers improve the overall strength and hardness through the recovery of kinetic energy, indicating that glass fibers have better ballistic properties.

In 2022, Chang and Zhang et al.^10^ combined the optimal injection parameter factors of the Pareto curve obtained by multi-objective optimization to verify and simulate the internal IME chip part of the UAV and showed that the correlation between the molding quality and injection molding pressurization time and mold index was higher than that of the other parameters. The IME film of the improved process meets the molding requirements of the core chip. In 2022, Chang and Zhang et al.^11^ used sequential approximation radial basis functions to optimize polymer injection molded automotive pedal defects. The Pareto boundaries were used to identify the warpage values over the cooldown time. Finally, the curve experimental data that meet the actual production requirements are given. In 2020, YoonLee et al.^12^ studied in-mold electronic technology conductive inks and completely filled the ink filler after high temperature and high pressure changes to improve the conductivity of the ink. Moreover, they performed the injection molding of optical devices and accurately displayed the circuit pattern. In 2020, Gong et al.^13^ studied the relationship between the resistance of thermal imaging IME molding and the deformation of the printed circuit, compared the deformation defect with the simulation results, and predicted the resistance distribution and size of the IME thermoforming process circuit. In 2020, Guo et al.^14^ combined microporous injection molding with IMD molding to achieve better appearance by changing the mold heat transfer simulation. Therefore, by performing a comparative analysis, the response model can predict the temperature field. This shows that the influence of the film on the molded part has a crystallization phenomenon under the condition of a large temperature change.

In 2020, Cheng^15^ designed the new film molding, considering some factors affecting the thermoforming and molding process in the experiment. Thus, the mold temperature, injection speed and holding pressure were only studied as injection parameters. Further optimization of the injection parameters showed that the parameter that had the greatest impact on the result was the holding pressure. The resistance measured after local hot pressing of the conductive wire in the surface mold is consistent.

In 2021, Liu et al.^16^ proposed a method of ink treatment based on broadband ultraviolet light, which can form traces with excellent properties compared with traditional hot sintering, while the production time is higher than that of the traditional method. Ink can be utilized to produce conductive silver from oxalate molecules on substrates and to develop interfaces that break traditional thermoforming. In 2021, Moayyedian et al.^17^ used a combination of Taguchi experiments to solve for the parameters of injection molding. The weight value of the factor of the part was calculated by layering analysis to simulate the polypropylene injection molded part, and the proposed optimum was verified. These injection parameters meet the actual requirements of the part. Moreover, the filling time has a significant impact on the finished product and molding quality. The error of the uncontrollable injection parameters during the injection molding process is 2.5%. In 2021, Chang and Zhang et al.^18^ proposed a hybrid screw process parameter method. To meet the requirements of production, a combination of an automated metering system and a performance evaluation program is proposed. The method predicts the injection of screws by extracting a combination of cutting conditions and related process parameters from the sensor data. Then, the sampling inspection methods were performed with the measurements obtained from routine inspection procedures.

**Figure 1:**
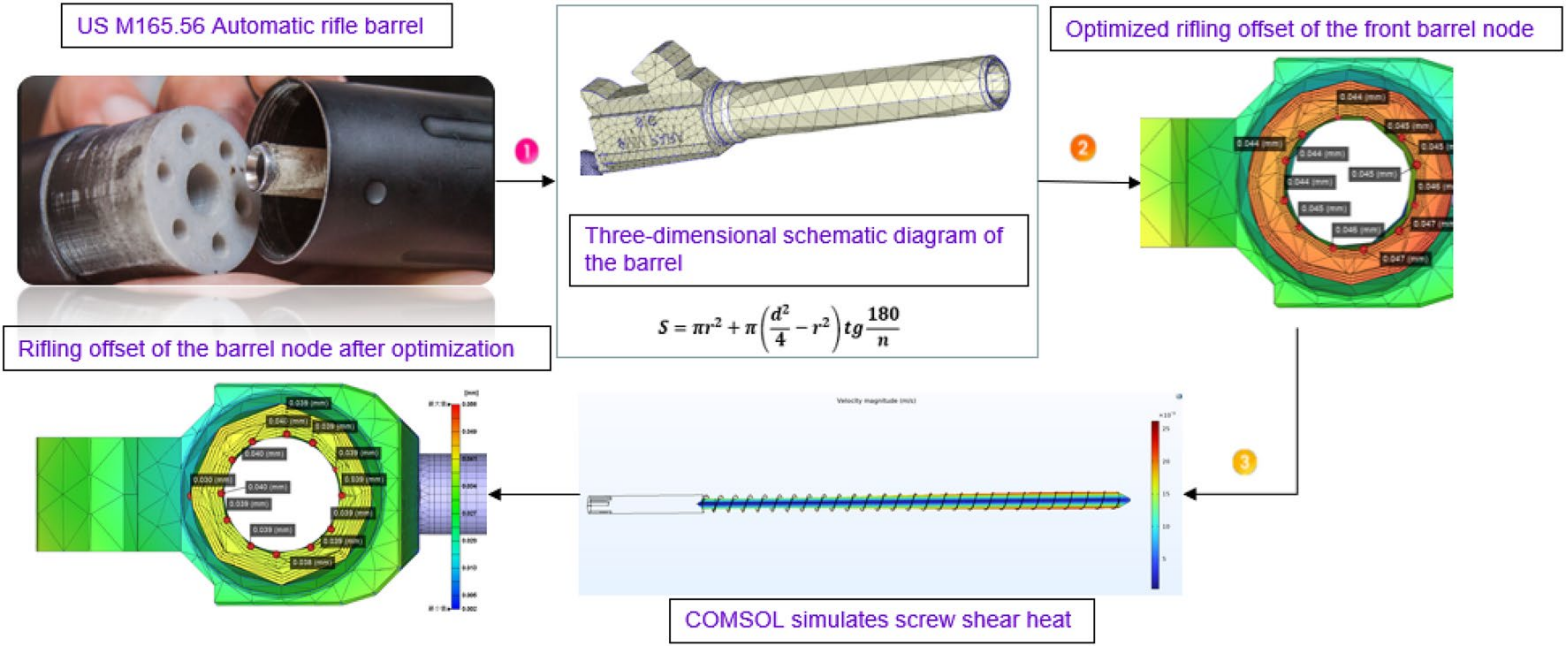
Overall structure.

At present, rifling processing is more studied than rifling design because the friction coefficient between the bullet and the barrel hole is not constant. Thus, the bullet movement time is short, and the deformation speed is fast, which makes the rifling force change and rifling shift experimental method in the rifling difficult to study. IMD is an efficient and cost-effective technique for setting one or even more layers of film between a film and a polymer material. The film is first placed in the mold, and the molten plastic is injected into the film. The film and plastic are combined into a single unit embedded in it. IMD can predict changes in the forming position on the surface of simulated parts, also known as offsets. The manufacturing process involves many variations in the injection molding parameters, such as the injection time, holding pressure, and cooling time. The manufacturing parameters are not optimally set or optimally combined, and the offset of the barrel rifling will have a large error. IMD-based nodal technology mold flow simulation and nodal simulation can be performed to solve for position changes in the experiments, which will be used to simulate the temperature stress changes and offset changes during rifling when attaching thin films to part surfaces. The overall structure of the article is shown in Fig. 1.

## Research methodology

### Rifling design method

**Figure 2:**
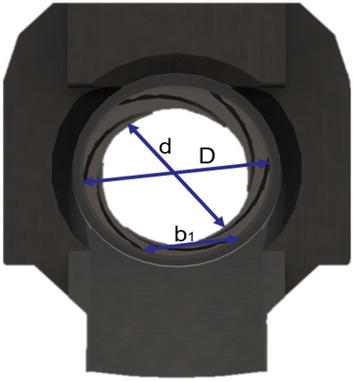
Schematic diagram of the barrel rifled cross-section.

The design is based on the breech structure analysis and selection and the breech size determination. The rectangular rifling is shown in Fig. 2. The advantages of rectangular rifling are that it is the simplest structure, can be conveniently processed and has a low cost. The disadvantage is that because the inner angle of the yin line and the outer angle of the yang line are similar to right angles, the rifling shell metal is not easy to fill when the rifling is embedded, which affects the strength at the intersection of the yin and yang lines. Moreover, the air closure is poor. During squeezing and steering, stress concentration easily occurs at the right angles, which reduces the strength of the barrel bore. At present, this structure is mostly used in the rifling of China's standard weapons. Figure 2 shows a schematic diagram of the barrel rifled cross-section.

The inner diameter of the rifling is called the caliber of the weapon, generally expressed in d. The D-brooming negative diameter is generally equal to the warhead cylindrical diameter zd, i.e.:

$$S=\frac{\pi {d}^{2}}{4}+n{b}_{1}h$$

For rectangular rifling, dz/d = 1.03 ~ 1.04, and the rifling depth h is half the difference between the diameter of the yin and yang lines. When designing new guns with existing firearms, the rifling depth h is fixed. When both the gun and the bullet are newly designed, their values should be determined in conjunction, and the following analysis should be considered. The value of dz/d is small, and the embedding force needed for the bullet to be embedded in the bore is also small, which is beneficial for improving the force condition of the ramp bore. However, a small h-value also reduces the area of the guide side. This will increase the rifling side stress, which is detrimental to the life of the barrel and does not effectively rotate the bullet. When dz/d is large, the opposite of the above situation occurs.

$$S=\pi {r}^{2}+\pi \left(\frac{{d}^{2}}{4}-{r}^{2}\right)tg\frac{180}{n}$$

The rifling number n is determined by tool (extrusion punch) manufacturing and measurement, and the rifling number n is generally taken as an even number. The positive line width b and the negative line width 1b have the following approximate relationship between the rectangular rifling: when the caliber d and the rifling number n are determined, the sum of the yang line and the yin line widths is approximately constant, and the rifling size is determined.

The rifling depth t should be chosen appropriately. Deeper rifling is beneficial to increase the barrel life but increases the amount of deformation of the projectile embedded in the chamber, making the maximum chamber pressure too high. At the same time, rifling that is too deep also easily causes the bullet to be broken by armor. Too shallow rifling affects the life of the body tube and reduces the warhead's steering ability, which affects the flight stability of the warhead. The relationship between the rifling depth t and the diameter of the black and white candlesticks is as follows: in Formulas (1) and (2), D is the diameter of the black line, and d is the white candlestick diameter.

**Figure 3:**
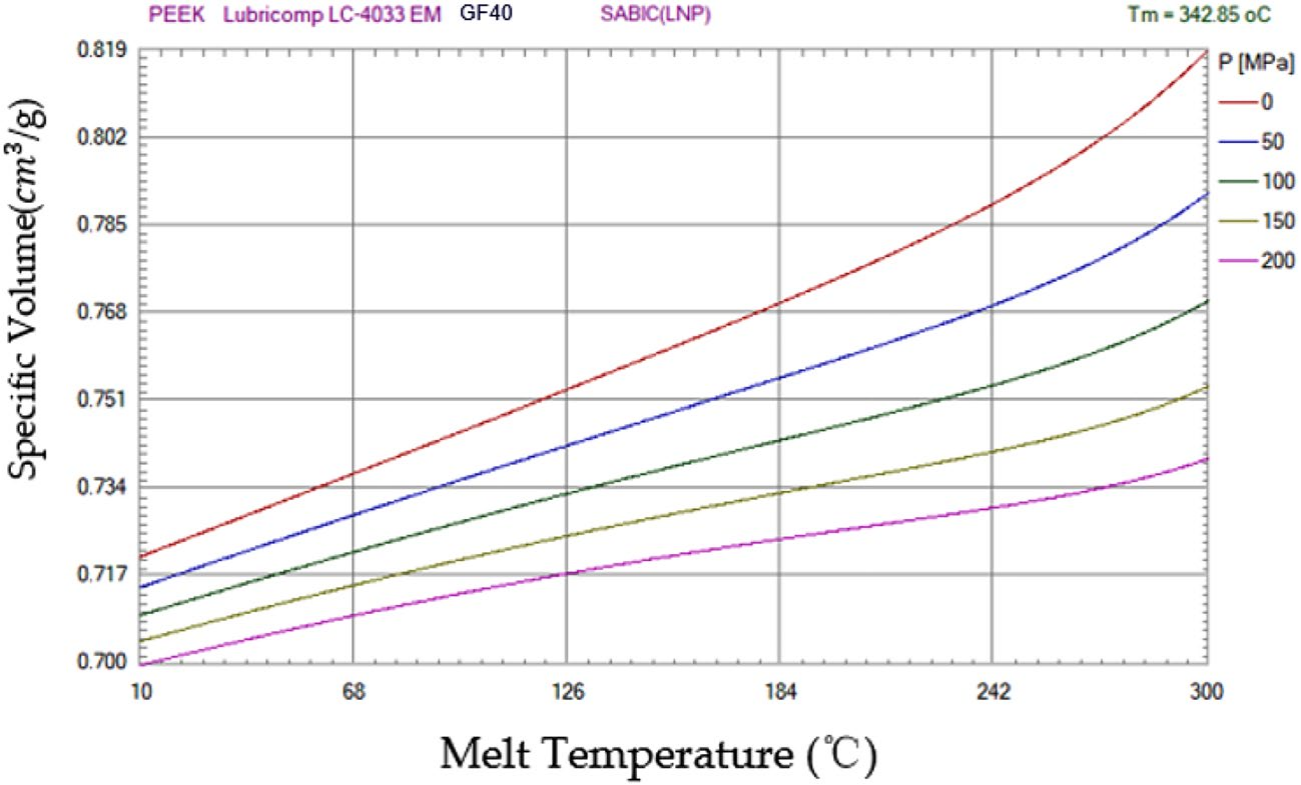
PEEK material PVT diagram (Moldex3D, https://www.moldex3d.com/).

According to experience, i = (0.01 ~ 0.02)d is generally used, where the upper limit value is implemented for small-caliber automatic weapons and the lower limit value is implemented for large-caliber automatic weapons. The number of white (or black) candlesticks on the inner chamber cross-section is called the rifling number and is represented by n. When rifling is shallow, the rifling number must be increased accordingly to ensure sufficient area on the conduction side. To facilitate the processing and measurement of rifling, the rifling number is usually taken as multiples of 4 or 3, such as 6, 24, 28 and 32. The rifling number increases with increasing caliber, with rifles having more than 4 rifling events and American 175 mm cannons having 48. In general, n is determined by rounding according to an empirical formula. Regarding infantry automatic weapons, the rifling number is chosen to be integers with multiples of 2. In the formula, d indicates the weapon caliber (mm), usually a 6–9 mm caliber body tube with a rifling number of n = 4 ~ 6 and 11–15 mm caliber body tube with a rifling number of n = 8. Different types of barrel property pairs are shown in Table 1. For example, in modern automatic weapons, the 7.62-mm caliber firearms have 4 bullets, and the 12.7-mm and 14.5-mm anti-aircraft machine guns have 8 bullets. Moreover, n = (3 ~ 5) d/10, where d is the caliber that is represented by mm, and n = 8d or n = πd(a + b), where d is denoted by in. A schematic diagram of the actual rifling of the barrel is shown in Figure 3.

These stress and strain values loaded during barrel production, derived from the rifling formula, will be converted into real stresses and real strains under real conditions in barrel rifling production according to JC experimental empirical numerical methods. Optical comparison is performed by comparing sequence images during specimen loading, providing precise displacement and strain fields on the rifling surface. Only one sensor is needed for in-plane measurements, but two sensors are needed for out-of-plane displacement. The equation also takes into account the relative displacement outside the plane of the offset region calculated by the barrel, so the strain in the area of the center of the gap can be provided.

When analyzing the barrel structure before the experiment, data on the material behavior under deformation and high strain of the fiberglass material during loading should be obtained. The combined simulation of the flow behavior of glass fiber materials based on the Johnson-Cook stress model was used for modeling and prediction. The material parameters of the PEEK flow stress are the temperature and PVT diagram of the material determined by tests performed within a certain strain rate range. It is proposed to combine the calculation parameters of the model specimen with the injection mold flow simulation to determine the damage parameters of the material failure model. Using these material parameters, the change model of the barrel offset under steady-state load was studied by a nodal offset test.

### IMD thin film technology

To solve the problem of excessive displacement of yin and yang line deviations during processing, a method to study mold indicators combined with IMD film is proposed, and the tensile properties and bending properties of the film itself are proposed. When setting its parameters, attention should be given to the influence of the diaphragm shape, blank clamping force, etc. Regarding the depth to avoid deformation, the thickness of the cylinder should be 2 mm when designing the film thickness, and when solving the stagnant temperature caused by the plastic film, the temperature difference of heat transfer can be calculated by cooling. Moreover, the amount of deformation can be minimized by compensating for the mold temperature on the mold side.

IMD is an efficient and cost-effective technique for attaching film to the surface of a part, which, unlike traditional printing, places a label between the film and resin. The printed film is placed in a mold, and molten plastic is injected into the film. The film and plastic are then combined into a single unit, and the decoration is embedded in it. Compared to other methods, IMD can improve the appearance and durability of the finished product. There are many parameters involved in the manufacturing process of IMD. If these manufacturing parameters are not set properly, the finished product is prone to defects. In current practice, the IMD parameters are determined based on trial and error or the personal experience of domain engineers, which can easily affect the yield of the IMD. Therefore, setting the parameters is a challenging task. This can be attributed to the following reasons. The position with the largest offset is mainly distributed in the center of the part, and it is also the part with the smallest thickness of the entire part and the part with the largest change in the mold index. It is necessary to analyze and improve the defects of this part. The IMD analysis results are optimized before and after the chip parts are produced, and the mold metrics are determined before and after IMD mesh analysis and film parameter optimization. Due to the excessive displacement of yin and yang line deviations during traditional processing, the proposed method of combining IMD film to study the die index is used to study the tensile properties, thickness, bending properties and elongation properties of the film itself. When setting its parameters, attention should be given to the influence of the diaphragm shape, die index corner radius, blank clamping force, mold gap, etc., on the drawing depth. To avoid product deformation when designing the film thickness, the barrel thickness should be 2 mm according to the analysis. When solving the stagnant temperature caused by the plastic film, the temperature difference for heat transfer can be calculated by cooling analysis, and the amount of deformation is minimized by compensating for the mold temperature on the mold side. IMD technology will be used to attach a thin film to the surface of the part.

In film molding quality research, the molding of plastic film should first be analyzed. During the in-mold IMD decoration process, the PEEK film in the experiment is put into the mold cavity before molding. Then, the same PEEK polymer material is melted into the mold cavity and combined with the film. Moreover, the molding film can be permanently glued to the surface of the plastic product after leaving the carrier film. When the melt is cured, after the mold is ejected from the product, a better decorative effect can be obtained, and the carrier will automatically peel off to prepare for the next production cycle. The film and the injection mold should match each other, the dimensions of the two should have high processing accuracy, and attention should be given to avoid the film in the turning position according to the extension of the film.

## Case study

The thickness of the initial material and the thickness of the offset at the groove on the barrel line are analyzed. When analyzing the structure before the experiment, data on the behavior of the material under deformation and high strain during flow are obtained. In this experiment, the combined simulation of the flow behavior of glass fiber materials was used for modeling and prediction. The PEEK flow parameter is the temperature, and the PVT plot of the material is determined by testing within a certain strain rate range. Therefore, it is recommended to combine the calculation parameters of the model with injection-mold flow simulation. Using these material parameters, the change model of cylinder offset under steady-state load was studied by a nodal offset experiment.

Composites are currently the fastest growing and most attractive ballistic building materials. They are the basic raw materials used in the production of composite bulletproof covers, such as shrapnel and bulletproof helmets, transport rifles, bulletproof covers, explosion protection devices, shrapnel and additional bulletproof inserts for bulletproof vests. The term "composite" refers to heterogeneous material structures. Composites can consist of a variety of metallic and nonmetallic materials used in combination. By selecting the right components, composites with the desired properties can be obtained, resulting in a lower weight, higher impact strength and better energy absorption. These properties are severely affected if the composite material exhibits defects (in the process and subsequent use), such as interlayer separation, air bubbles and delamination, which are usually caused by impact. The authors propose a thermal model of a composite that introduces defects. Thus, a series of computer simulations can be conducted to locate inclusions, the composite can be reduced, a simple Level I repair of the overlay can be performed, and the material filling can be 3D-printed from recycled pad for repair. Moreover, the pad is repaired to reinforce the strength of bullet rifling. The overlay is added to the panels by lamination technology, followed by the use of vacuum bags to provide sufficient compaction pressure. Secondary repairs and tertiary repairs require more materials, and they are still being studied. In rifled and body armor systems, the fiber diameter, metal surface stiffness, areal density of woven fabrics and bulk density of glass fibers are key parameters that will form the basis of relevant quality control procedures. The interface that exists between the various products is often overlooked, since it may belong to the domain of the user.

Plug-in (SAI) and one or two hard bullet rifling plates (HAP1 and HAP2) are studied. This is especially true if different products come from different suppliers. A typical human bullet rifling system has between 30 and 150 individual layers, and each interface between each layer affects the ballistic performance of the system in some way. For example, the following interface/mezzanine parameters are considered: (i) friction, slip, and interlaminar surfaces within the soft bullet rifling assembly and between the component and the carrier, (ii) air gaps that may form within the soft bullet rifling assembly, (iii) interconnection space between the soft bullet rifling assemblies and hard bullet rifling plates, (iv) interfacial properties between the adjacent layers of multilayer backing layers even in highly compressed ultrahigh molecular weight polyethylene (UHMWPE) variants, and (v) interlayers between glass fibers within HAP and their substrates, and (vi) the geometric fit between the two hard shields in the stacked human protection system. When performing breakage offset detection, it is necessary to perform corresponding offset detection operations at the interface, obtain the corresponding current and voltage values, and then perform comparative analysis to obtain the final offset detection result of the ability and formability, widely used in the manufacture of rifled vehicles to protect against external threats.

These materials can experience a variety of strains, temperatures, and pressures when subjected to dynamic loading conditions, such as ballistic impacts. The area with a large die index is an area that is easily damaged, and the possibility of damage is increased, which provides a damage prediction function for the warframe. For the damaged warframe, this method can also be used for offset detection because the damaged part is more likely to cause the surface film to rupture after impact, so it can be used through the die index to optimize the design and molding of the warframe. Moreover, the offset can be used to detect the damage degree of the damaged part of the damaged part of the machine. Therefore, the IMD die index is introduced to quantify the data, and the rifled glass fiber material is a very promising material that combines high strength and high ductility with excellent load bearing.

**Figure 4:**
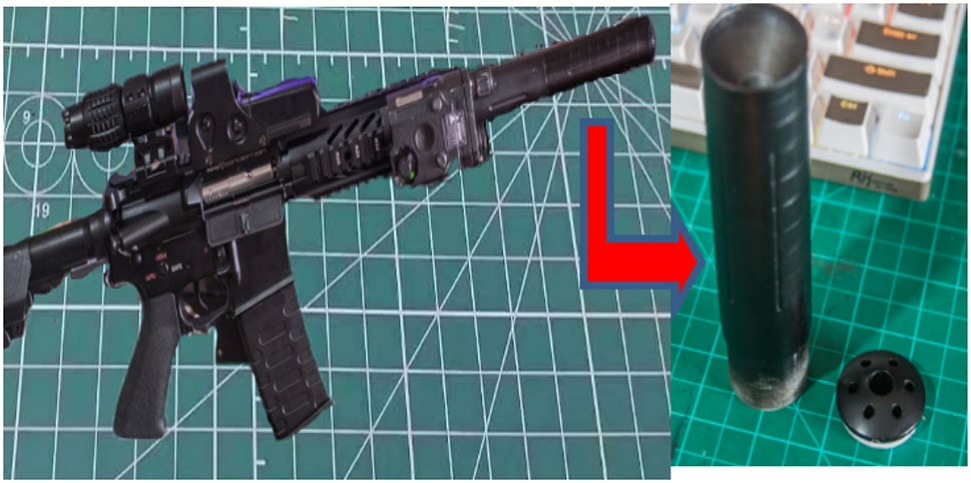
US M165.56 automatic rifle barrel assembly drawing.

The viscoelastic plastic model of the PEEK separator and the PVT diagram of the PEEK material in Film 3 were constructed theoretically, and the elastoplastic part of the PEEK separator during deformation was characterized. In this experiment, the automatic PEEK plastic film of Fig. 4 US M165.56, which had the characteristics of good dimensional stability at high temperature, high temperature resistance and friction resistance, was used. According to the solid model of the composite in previous studies, the characteristics and stress of the PEEK polymer are reflected.

**Figure 5:**
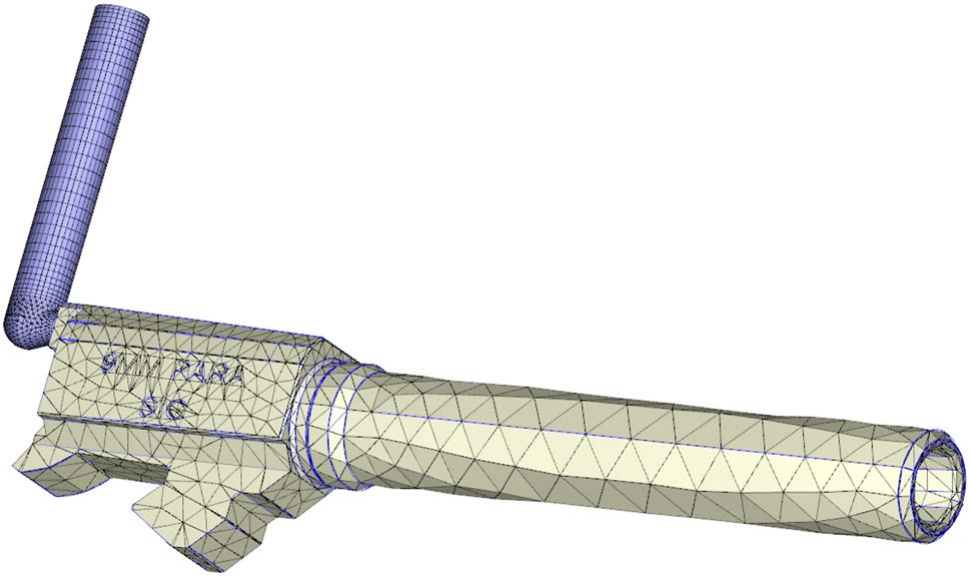
Three-dimensional schematic diagram of the barrel (Moldex3D, https://www.moldex3d.com/).

In this experiment, 6 groups of gun lines are divided into 12 node measurement offset lines, and the actual barrel of the gun is shown in Fig. 5. Then, 5 nodes in each offset line are selected for deviation measurement, with the deviation value of the node and the law being focused on. Then, the gun line deviation value obtained by the design of this experiment is compared with the traditional processing gun line deviation value, reflecting the superiority of the node deviation method and the accuracy of the PEEK material barrel line position.

**Figure 6:**
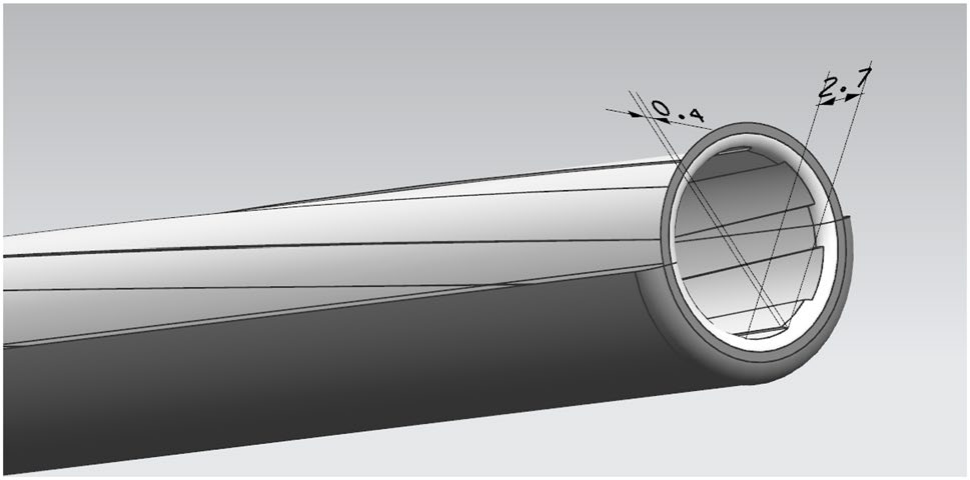
Drawing of the rifled dimensions of the barrel.

The mesh is the hexahedral normal mesh of the subdivision criterion, and the mesh is repaired to obtain a completely convergent result, as shown in Fig. 5. The strain distribution of the material in the neck is almost uniform, while the stress state in the center of the notch is triaxial. Moreover, offset initiation always occurs. Bridgman performed a stress analysis on these specimens, and he provided a three-axis equation for the centers of these specimens, where R is the radius of the curvature of the neck. The initial stress triaxial of the round specimen is 1/3. Different triaxial properties can be obtained by creating notches of different radii. This equation can be used to predict the higher stress triaxial nature of the gap with lower radii. Schematic Fig. 6 of the barrel rifling is shown below.

These equations are often used for round specimens to determine the triaxiality effect for failure models in armor steels. However, this formula is limited only for round specimens and is not capable of predicting the triaxiality for flat specimens such as hot-rolled amour plates, which are difficult to machine into cylindrical specimens. Such sheets are widely used in automotive, aerospace, and defense applications. Very few studies are available where flat specimens were used to study the triaxiality effect. This paper proposed the triaxiality at the center of the notched region for a plane strain specimen, where R is the radius of the notch and t is the specimen thickness at the notched region.

**Figure 7:**
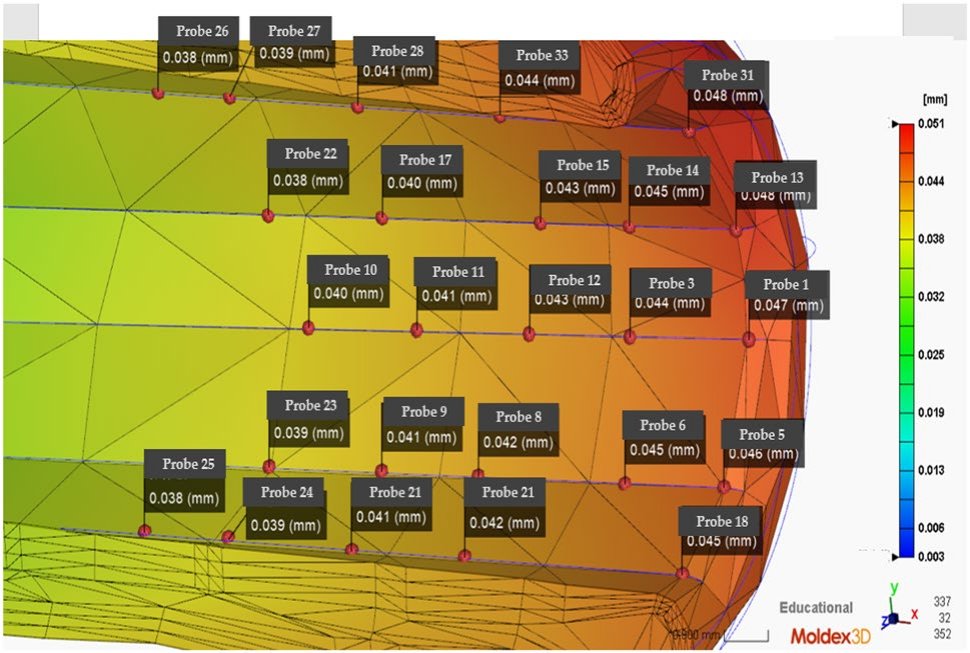
Initial distribution of the barrel rifling nodes (Moldex3D, https://www.moldex3d.com/).

In practical engineering, it is difficult to calculate rifling deviations directly, and the finite node difference method is the most common and efficient method for solving partial differential equations. The barrel wall thickness and time are discretized. The nodes are used, and the inner wall of the barrel undergoes two processes: heating and cooling. Moreover, the corresponding nodal difference equations are established. The offset data for the nodes are shown in Fig. 7, and the data are summarized in Table 2.

**Table 2:** Moldex simulation results for the die index matching table.

Number of nodes	Right die Index (mm)	Left die Index (mm)
A1	0.083	0.052
A2	0.046	0.056
B1	0.054	0.043
B2	0.051	0.030
C1	0.044	0.039
C2	0.078	0.021
D1	0.049	0.048
D2	0.038	0.012
E1	0.046	0.056
E2	0.054	0.043
F1	0.051	0.030
F2	0.044	0.039

Obvious offset errors occur in Groups A1, B2 and C2, reaching deviation values outside the error range, and the injection molding parameters need to be optimized first. There are many parameters involved in the manufacturing process of IMD. If these manufacturing parameters are set incorrectly, the finished product can easily become defective. Specifically, defects in the IMD are concentrated at end locations. The figure shows the mold index for the left and right nodes of two groups of six nodes representing the damaged area.

## Result

**Figure 8:**
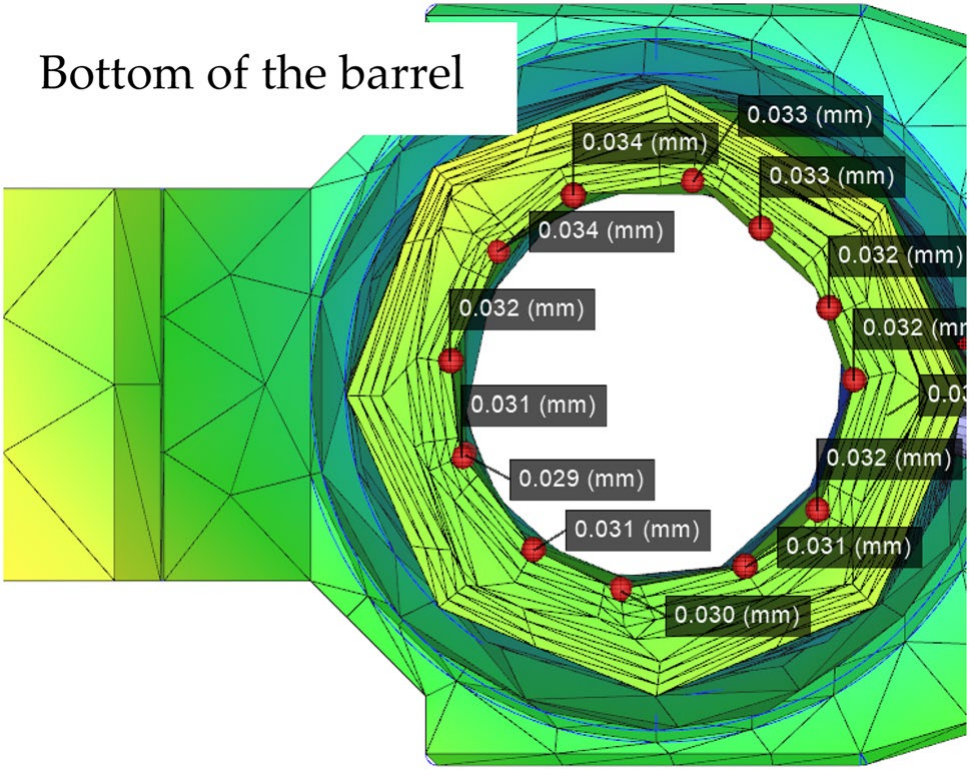
Numerical plot of the bottom of the barrel at the rifling node (Moldex3D, https://www.moldex3d.com/).

The fiberglass material used in this study is a 3-mm thick barrel as well as a 0.5 mm deep wire that flattens the barrel experimental subject according to the standard geometry. Then, tests on the subdimensions of the barrel are performed. Figure 8 shows the subsequent tests. Injection molding is performed to optimize the subsequent parameters. The changes in the barrel rifling offsets for different sol temperatures, temperatures and depths are shown in Figs, 8, 9 and 10, respectively.

**Figure 9:**
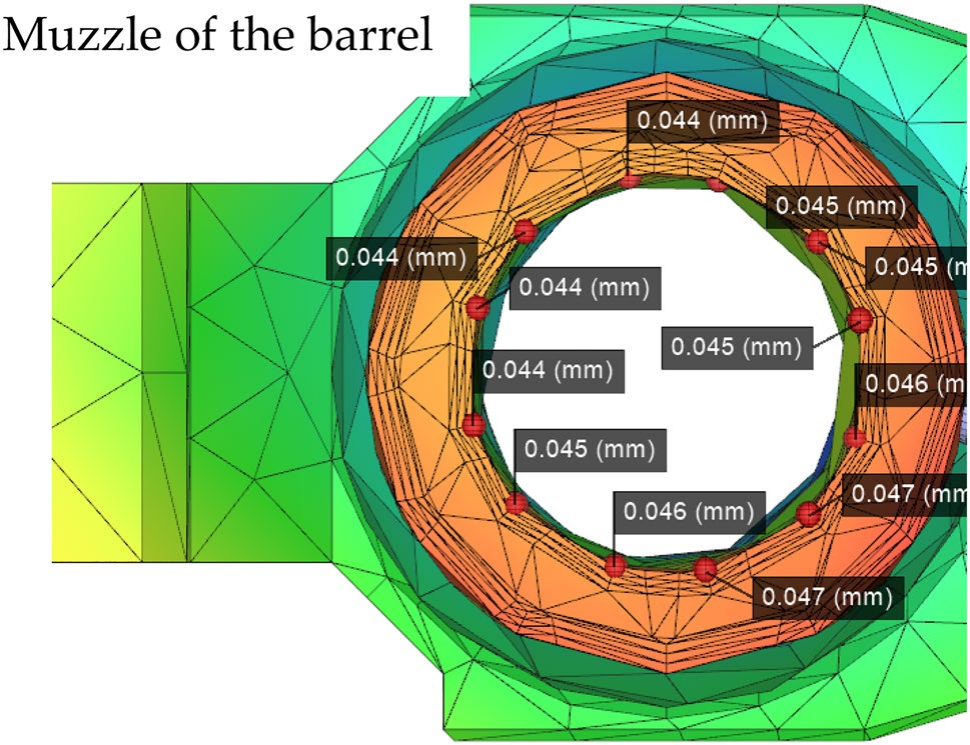
Numerical plot of the rifled node muzzle of the barrel (Moldex3D, https://www.moldex3d.com/).

**Figure 10:**
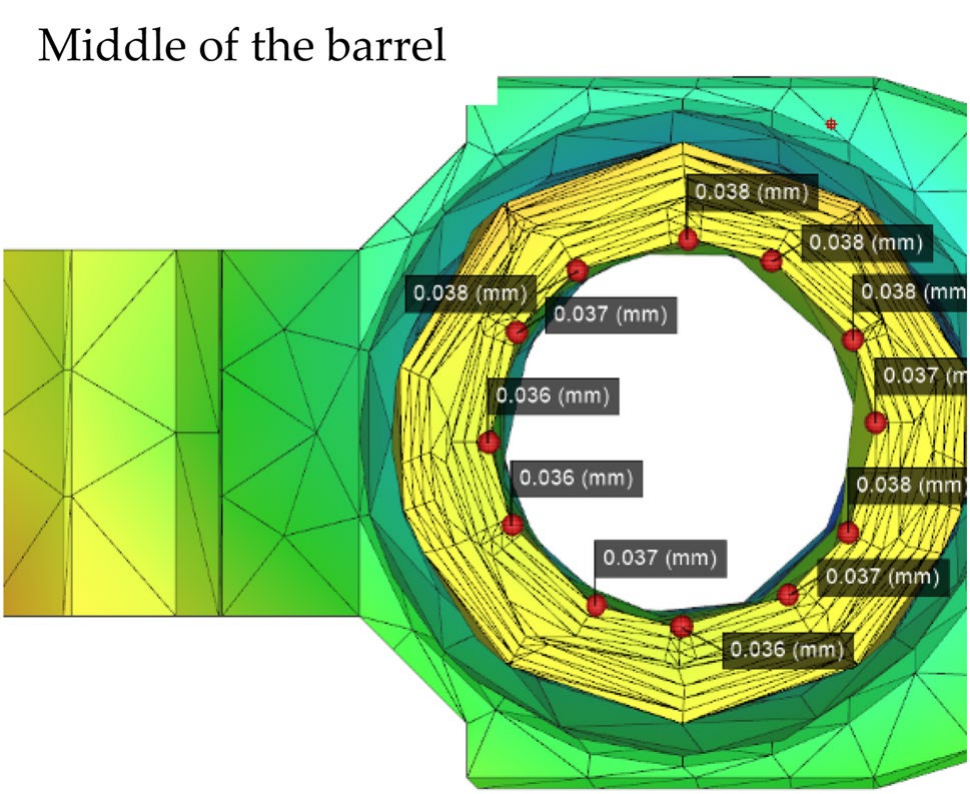
Numerical plot of the middle barrel of the rifled node (Moldex3D, https://www.moldex3d.com/).

The experiment is carried out through the needed warpage value and die index response surface analysis, as well as the injection molding parameters holding time, filling time and clamping force mathematical relationship. Then, the multi-objective optimization program analysis experiment is performed. The overall parameters are set, the multi-objective method of sequential approximation optimization is used for optimization, floating-point encoding is used to set the overall size of the algorithm to 100, the evolutionary algebra is 100, 200, 200, 300, the crossing probability is 0.9, and the mutation probability is 0.1. Through the above experiment, the multi-objective optimization analysis of the population is carried out. Then, the analysis is conducted to verify whether the data point values truly meet the optimization results and to obtain the fit and fitness evaluation of the results of the experiment. The adaptability assessment is a measure of the quality of a solution, and it is often based on the relationship between the solution's behavior and the environment or population. It is usually represented by the objective function, and the fitness value of the solution is the main basis for selection during evolution. In the evolutionary search process, external information is basically not used, and only the fitness function is used as a basis to search by the fitness value of each individual in the population. Fitness directly affects the rate of convergence and whether it can be found. In the early stage of genetic evolution, some abnormalities usually appear. If the proportional selection method is adopted, these abnormalities affect the selection process due to their strong competitiveness, which affects the overall optimization performance of the algorithm. In the selection process, that is, when the algorithm is close to convergence, the possibility of continuous optimization is reduced because the individual adaptability difference in the population is small, and the three sets of optimal parameters obtained after the optimization result of the local optimal solution can be obtained are shown in Table 3.

**Table 3:** Moldex injection parameter data allocation table.

Multi-segment packing data	Split time interval (s)	Holding pressure (first stage)	Holding pressure (second stage)	Holding pressure (third stage)
Holding time (s)	0 ~ 4.15	100	90	60
	4.15 ~ 5.53	90	75	50
	5.53 ~ 6.92	80	60	40
Volume shrinkage	Unit of (%)	12.9	12.8	13.1
Maximum warpage value	Unit (mm)	0.78	0.58	0.36
Fi	Unit (s)	1.86	1.88	2.02
Maximum Cf	Unit (MPa)	23.86	21.05	13.7

**Figure 11:**
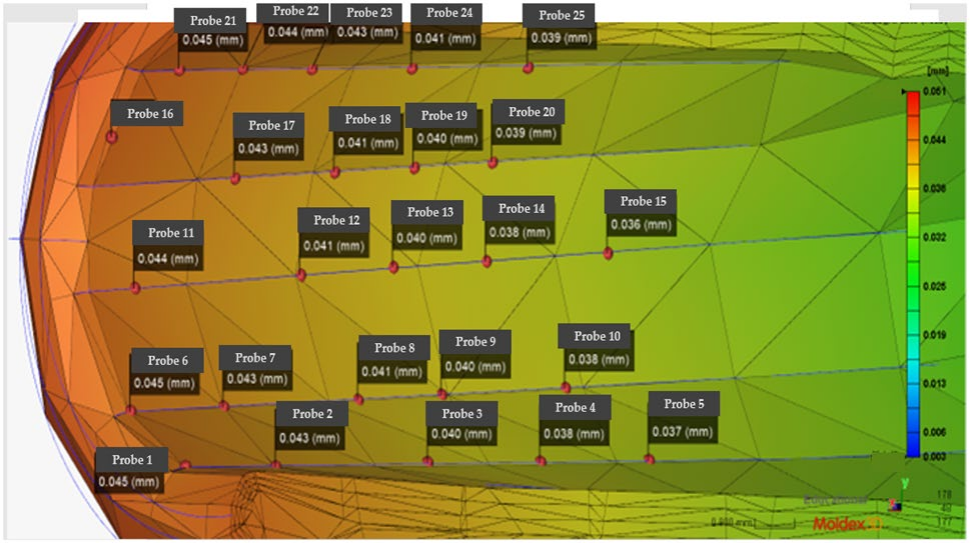
Barrel rifling node optimized die index diagram (Moldex3D, https://www.moldex3d.com/).

To verify the location of the damage, we also carried out the verification of the relevant load‒loading model in the COMSOL software. This model is used to make assumptions of the stress distribution, the load impact is simulated when the armor is damaged, and a damage map of the relevant maximum damaged position is obtained, as shown in Fig. 11. The results of the mold flow software are compared to verify the accuracy of the node technology. Table 4 has a summary of the nodes.

**Table 4:** Moldex optimizes the simulation results to the die index matching table.

Number of nodes	Right die index (mm)	Left die index (mm)
A1	0.053	0.042
A2	0.056	0.053
B1	0.044	0.043
B2	0.031	0.030
C1	0.034	0.039
C2	0.028	0.021
D1	0.049	0.048
D2	0.038	0.012
E1	0.046	0.056
E2	0.054	0.043
F1	0.051	0.030
F2	0.044	0.039

It can be seen from the analysis results that the actual degree of barrel offset is positively correlated with the depth change, and as the depth increases, the offset will also decrease. The fiberglass material used in this study is a 3-mm thick barrel and a 0.5-mm deep gun wire, which can be flattened according to the standard geometry of the barrel experimental subject. Then, sub-dimensional testing of the barrel part geometry optimizes subsequent injection molding engineering parameters, varying filling times and inlet pressures and temperature changes.

**Figure 12:**
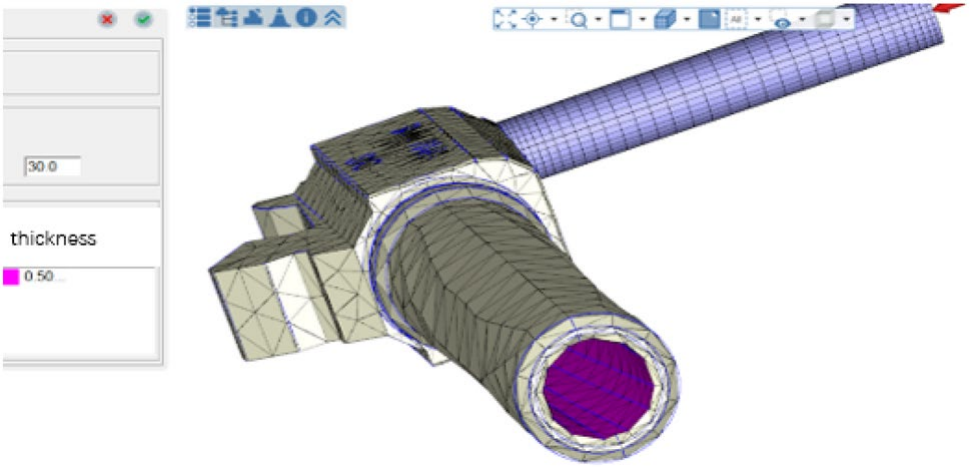
Numerical diagram of the IMD film inside the barrel (Moldex3D, https://www.moldex3d.com/).

From the analysis results, it can be seen that the actual offset degree of the barrel is positively correlated with the temperature change, and the offset decreases with increasing temperature. The Johnson–Cook offset prediction model is used to predict the material offset behavior of glass fiber, and the value of the IMD film inside the barrel is shown in Fig. 12. Then, the subsequent injection molding engineering parameters are tested and optimized on the sub-size of the barrel part geometry, and the corresponding simulation is performed on the MTLAB to obtain the relationship between the warpage and die index.

Since the temperature of this screw is affected by shear heat, the coupling physics of the screw is selected for non-isothermal rotating mechanical-laminar flow physics. Because of the PEK when the polymer fluid is under the flow dynamics of temperature field heating, its viscosity is still high, and the order of Reynolds number is estimated to be 10^−3^ according to the empirical formula. Therefore, laminar flow research is conducted. Additionally, because physical properties such as the density, constant pressure heat capacity, thermal conductivity and dynamic viscosity of the material are related to temperature, the physical model is set to weak compressible flow; that is, the density of the material is only related to temperature. The fluid flow equations and heat transfer equations in multi-physics are shown in Eqs. (1) and (2).

1 $$\uprho (\mathrm{T})(\mathrm{u}\cdot \nabla \mathrm{u}) =-\nabla \mathrm{p}+\upmu (\mathrm{T}) \nabla \mathrm{u}+\mathrm{ F}$$

2 $$\mathrm{\rho Cpu}\cdot \nabla \mathrm{T }+ \nabla \cdot (-\mathrm{ k}\nabla \mathrm{T}) =\mathrm{Q}$$

where ρ is the density of the material, kg/m^3^; T is the temperature, K; u is speed, mm/s; p is the pressure, Pa; μ is the expansion viscosity coefficient; F is the force, N; C_p_ is the constant pressure heat capacity, J/(kg K); k is the thermal conductivity W/(m^2^ K); and Q is the heat of the external heat sources.

**Figure 13:**
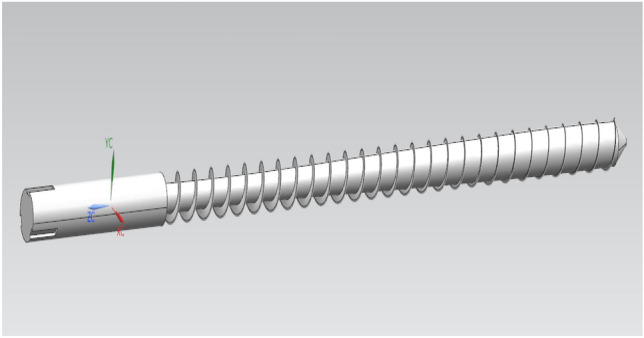
Three-dimensional schematic diagram of the screw.

The fluid flow velocity in the coupling interface is generated by laminar flow physics and inputted into the heat transfer equation. Moreover, the temperature in the coupling physics is generated by the fluid heat transfer physics and fed into the fluid flow equation to achieve a two-way coupling of the flow field and the heat transfer field. The density of the material is determined by the temperature output in the heat transfer field according to the density curve. Due to PEEK's high viscosity, the viscous dissipation term and the work term are considered in Q on the right side of the heat transfer Eq. (2). A three-dimensional schematic of the screw is shown in Fig. 13.

**Figure 14:**
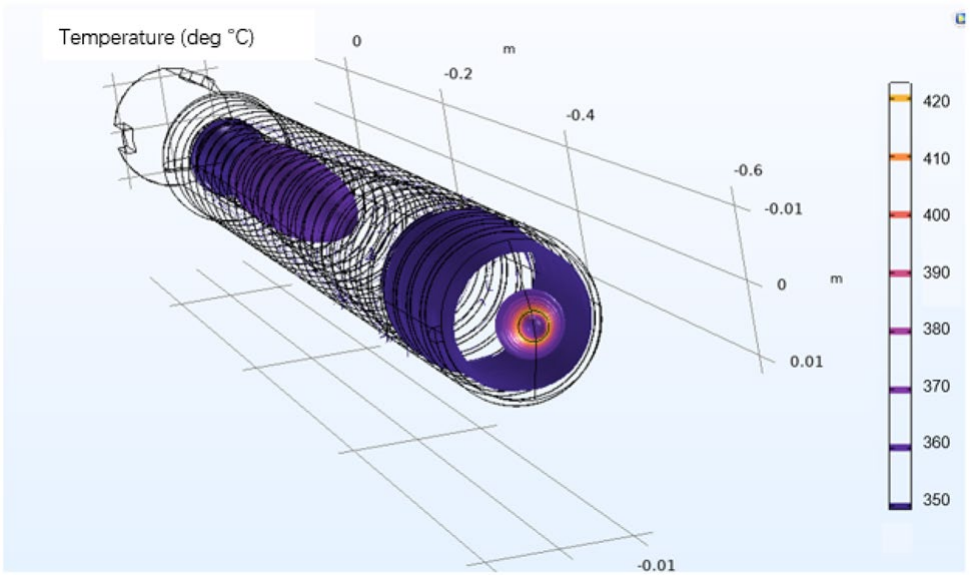
Screw motion heat generation equivalent surface temperature diagram (Comsol5.6, http://cn.COMSOL.com/).

**Figure 15:**
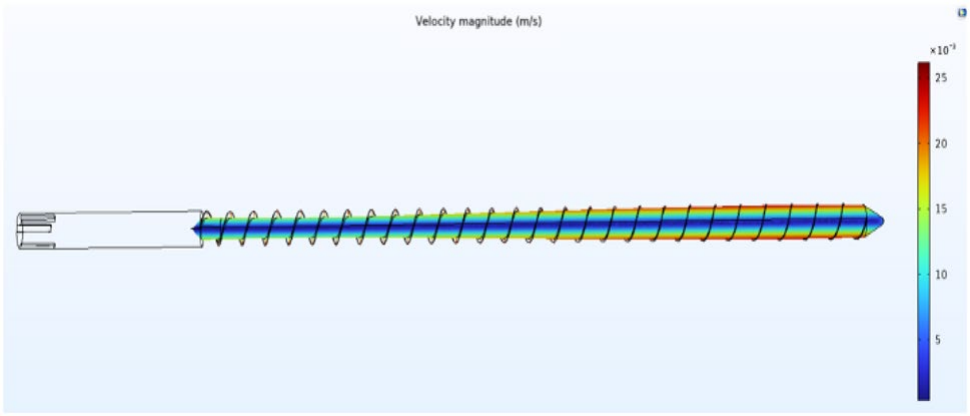
Diagram of the heat generated shear rate of the screw motion (Comsol5.6, http://cn.comsol.com/).

By studying the influence of the temperature, shear rate and other factors on the plasticization temperature of the PEEK screw material during processing, the PEEK processing process in the plasticizing unit of the screw microinjection molding machine was studied by a simulation method, and the flow, heat transfer and phase change of PEEK in the plasticizing process were simulated. The effect of the screw shear rate on the temperature was analyzed. The screw speed is set to a fixed value (10 r/s), and the phase transition situation is significantly improved as the temperature rises. However, the increase in speed and shear rate is not obvious, and only after the screw speed exceeds 50 r/s will there be a more obvious increase. When the heating temperature is set to a fixed value (400 °C), there is no significant change in the phase change, speed or shear rate as the screw speed increases. The temperature and shear rate of the heat generated by screw motion are shown in Figs. 14 and 15.

The dimensions of the screw are as follows: ∅25. L/D = 27, L1 = 12 D, L2 = 10 D, L3 = 5 D, h1 = 4. 1 mm, h2 = 3.8, h3 = 0.9 mm, w = 19.0 mm, e = 5 mm, n = 100 rpm, h3 = L3; gradient groove starting depth, h1 = initial depth of the L1 gradient groove, h2 = the starting depth of the L2 gradient groove, w = D = gradient slot width, e = axial thread width, n = rotation speed.

**Figure 16:**
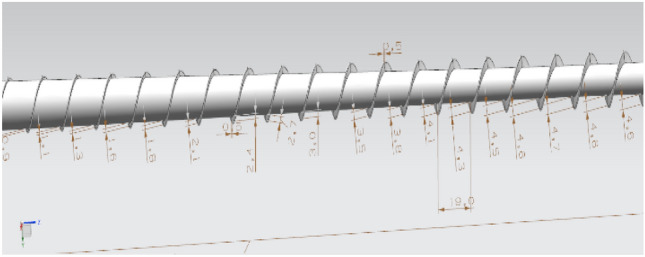
Schematic diagram of the screw size.

**Figure 17:**
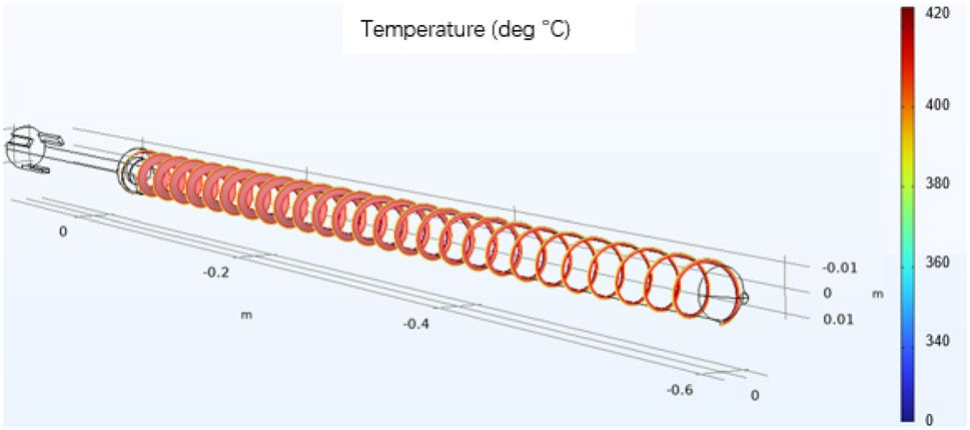
Diagram of the temperature of the movement of the outer end of the screw (Comsol5.6, http://cn.COMSOL.com/).

In this experiment, the depth node temperature comparison was compared by 10% in the size of the base tank depth h, and the actual size of the screw is shown in Fig. 16. The influence relationship between the increase in the shear heat and the change in the tank depth is determined. We used COMSOL 5.6 software to simulate the uniform rotation motion, and the temperature change of each section at the outer end of the screw is shown in Fig. 17. In this experiment, the depth node temperature was compared by reducing the size of the base tank depth h by 10%, with the influence relationship between the increase in the shear heat and the change in the tank depth being determined.

**Figure 18:**
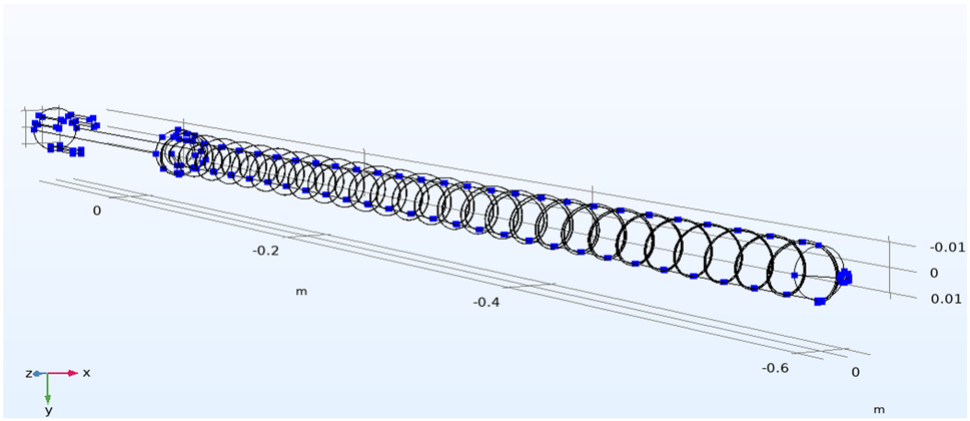
Screw node selection distribution map (Comsol5.6, http://cn.COMSOL.com/).

To verify the rifling offset position, the rotational motion model of the screw was simulated in COMSOL software, as shown in Fig. 18, and the assumption of stress distribution was made using this model theory. The offset model when the gun line was offset was simulated, and the relevant offset image was obtained, as shown in the figure. Then, the results of the mold flow software are compared to determine whether the two match to verify the accuracy of the node technology.

According to the temperature simulation results, we take two symmetrical nodes in each section of the outer surface of the screw for temperature detection, and according to the image simulation result of Fig. 18, we obtain the optimization effect diagram of the node offset value, and the specific values are presented in Table 3.

**Figure 19:**
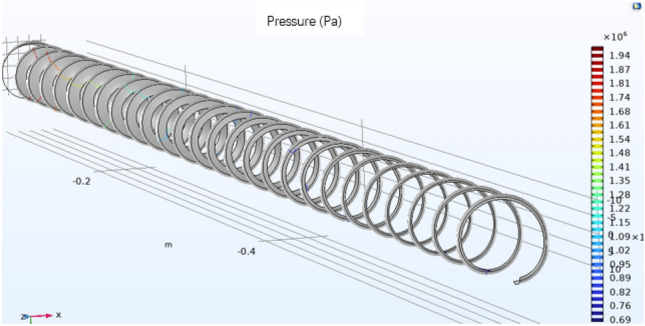
Contour diagram of the motion pressure at the outer end of the screw (Comsol5.6, http://cn.COMSOL.com/).

In this experiment, the width of the basic average groove width D = 19.0 mm is reduced by 10%, and the nodal temperature is compared to obtain the influence relationship between the increase in the shear heat and the change in the groove depth, as shown in Fig. 19. This figure shows the contour diagram of the pressure change at the outer end of the screw, and the comparison of the pressure change of the glue inlet can be determined by using the degree of pressure increase.

From the analysis results, it can be seen that the actual offset degree of the barrel is positively correlated with the temperature change, the offset decreases with increasing temperature, and the Johnson–Cook offset prediction model is used to predict the material offset behavior of glass fibers. Then, the subsequent injection molding engineering parameters are tested and optimized on the sub-size of the barrel part geometry, and the corresponding simulation is performed on MATLAB to obtain the relationship between warpage and die index.

## Discussion

**Figure 20:**
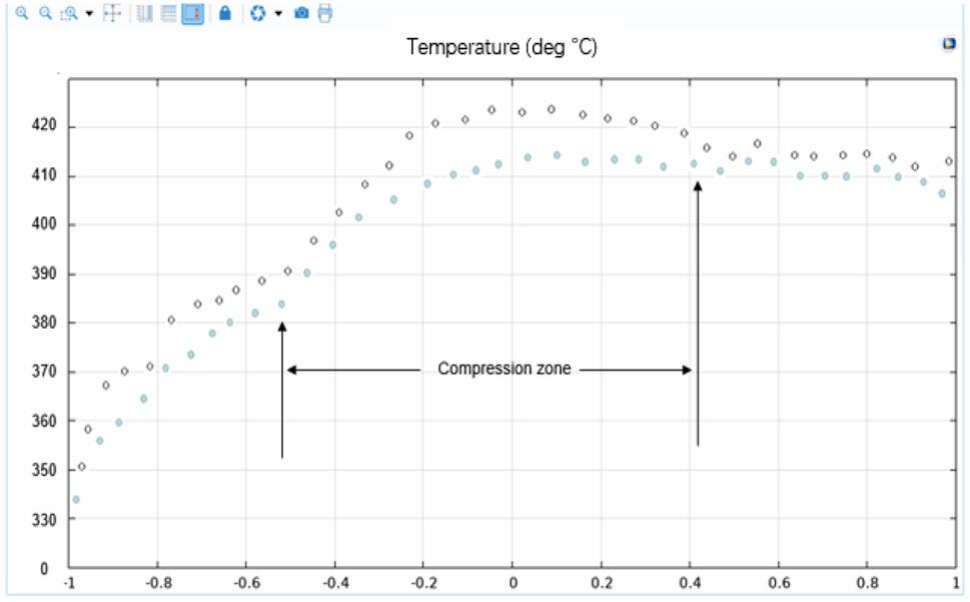
Numerical diagram of the node of the compression zone after the screw increases the groove depth h (Comsol5.6, http://cn.comsol.com/).

**Figure 21:**
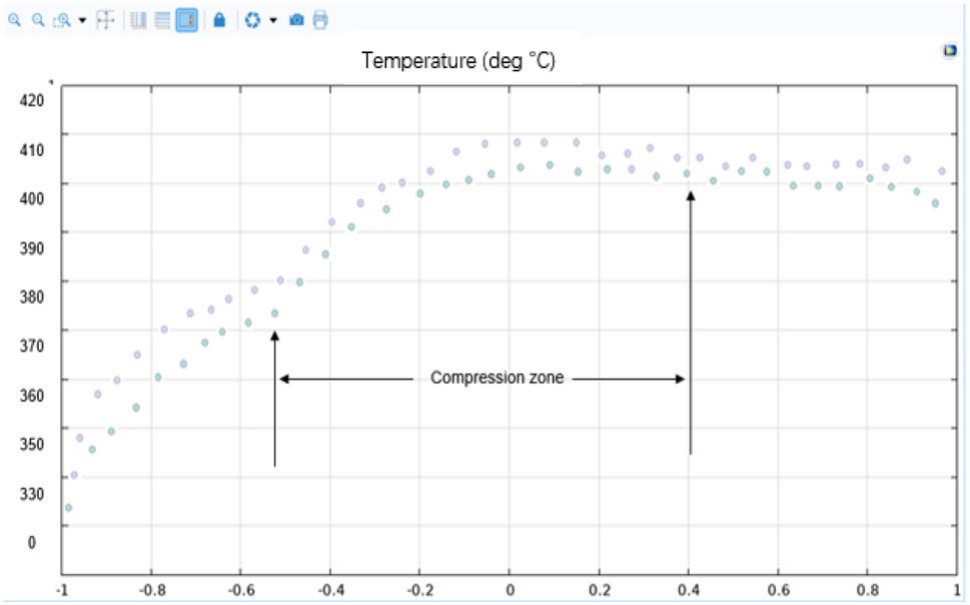
The width of the screw L/D = 29 numerical figure of the node in the compression zone (Comsol5.6, http://cn.comsol.com/).

By studying the influence of the temperature, shear rate and other factors on the plasticization temperature of the PEEK material screw during processing, the PEEK processing in the plasticizing unit of the screw microinjection molding machine was studied by a simulation method, and the actual degree of offset of the barrel is positively correlated with the depth change. The offset decreases as the depth increases. The glass fiber material used in this study is a 3 mm thick barrel and a 0.5 mm deep gunwire, which can be flattened according to the standard geometry of the barrel experimental subject and then tested on the subsize of the barrel part geometry to optimize the subsequent injection molding engineering parameters, changing the filling time and inlet pressure and temperature changes. The numerical plot of the nodes that change the filling time and the inlet pressure and temperature is shown in Figs. 20 and 21.

According to the image simulation results, we obtain the optimization effect of the node offset value, and the specific value is presented in Tables 5 and 6.

**Table 5:** COMSOL screw shear heat generation simulation results temperature index node numerical table.

Shear thermal contrast	Reduce the width of the rear screw L/D = 29
Initial screw size L/D = 27	Average shear heat rises ratio r = 0.45%
The screw after increasing the groove depth h	Average shear heat rises ratio r = 0.32%

**Table 6:** Moldex3D value table after optimizing the screw temperature.

Number of nodes	Temperature (L/D = 27)	Temperature (L/D = 29)	Rate (%)	Temperature (h = 5.5 mm)	Shear heat change rate (%)
1	389.7 °C	391.3℃	0.41	390.5 °C	0.41
2	396.8 °C	397.8℃	0.25	397.3 °C	0.18
3	402.3 °C	403.3℃	0.24	402.9 °C	0.21
4	408.6 °C	408.9℃	0.07	408.8 °C	0.05
5	411.7 °C	413.2℃	0.12	412.3 °C	0.09
6	414.6 °C	418.9℃	1.02	416.8 °C	0.89
7	419.8 °C	421.0℃	0.28	420.3 °C	0.18
8	420.5 °C	422.3℃	0.43	421.6 °C	0.23
9	418.0 °C	424.6℃	1.55	423.6 °C	1.25

**Figure 22:**
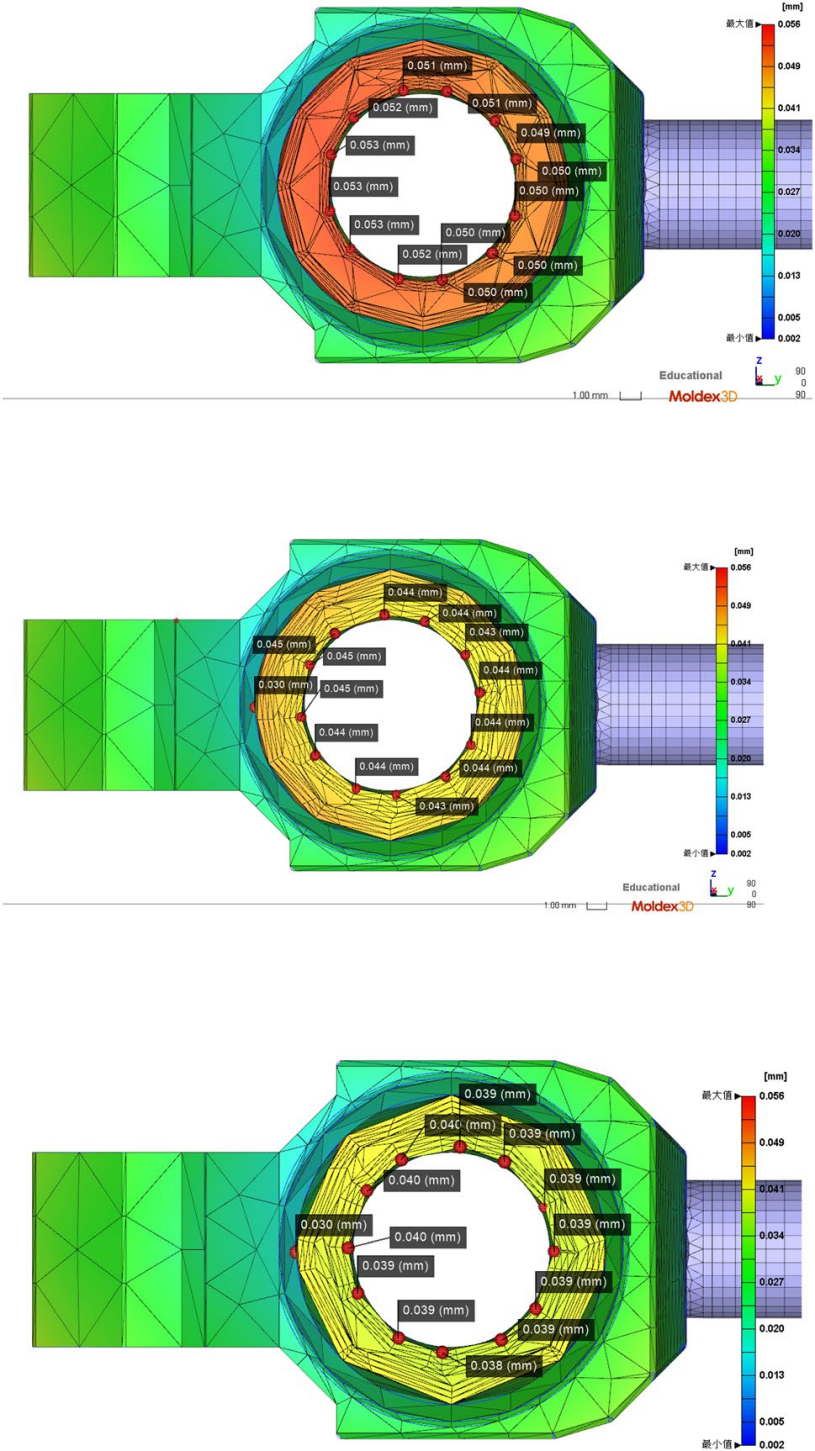
Numerical plot of Moldex3D after optimizing the screw temperature (Moldex3D, https://www.moldex3d.com/).

It can be seen from the analysis results that the temperature of the barrel will change after changing the groove depth and groove width, and in the same degree of size change, the influence ratio of groove depth is lower than that of the groove width. Moreover, the displacement offset image in Moldex3D is obtained by substituting the changed temperature. The actual degree of offset of the barrel is positively correlated with the depth change, and the offset decreases as the depth increases. The glass fiber material used in this study is a 3 mm thick barrel and a 0.5 mm deep gun wire, which can be flattened according to the standard geometry of the barrel experimental subject. Then, tests on the subsize of the barrel part geometry were performed to optimize the subsequent injection molding engineering parameters, changing the filling time, inlet pressure, and temperature, as shown in Fig. 22.

From the analysis results, it can be seen that the actual offset degree of the barrel is positively correlated with the temperature change, the offset decreases with increasing temperature, and the Johnson-Cook offset prediction model is used to predict the material offset behavior of glass fibers. Then, the subsequent injection molding engineering parameters are tested and optimized on the sub-size of the barrel part geometry, and the corresponding simulation is performed on MATLAB to obtain the relationship between the warpage and die index. In this experiment, the influence of the barrel temperature, shear rate and other factors on the plasticization temperature of the PEEK screw material in Fig. 21 of US M165.56 automatic rifle during the processing was studied, and the PEEK processing in the plasticizing unit of the screw injection molding machine was studied by a simulation method. The flow, heat transfer and phase change of PEEK in the plasticizing process were simulated, and the influence of the screw shear rate on temperature was analyzed. The screw speed is set to a fixed value (10 r/s), and the phase change is significantly improved as the temperature rises. However, the increase in the speed and shear rate is not obvious, and only after the screw speed exceeds 50 r/s will there be a significant increase. When the heating temperature is set to a fixed value (390 °C), there is no significant change in the phase change, speed or shear rate as the screw speed increases. The actual offset degree of the barrel is positively correlated with the depth change. With increasing depth, the offset will also decrease. The glass fiber material used in this study is a 3 mm thick barrel and a 0.5 mm deep gun wire, and the increase in the slot width will also lead to an increase in shear temperature, affecting the PEEK feed temperature and pressure. The final temperature change will affect the size of the barrel line offset. The higher the temperature is, the larger the offset value.

## Conclusion

In this experiment, the depth node temperature comparison was compared by 10% in the size of the base tank depth h, with the aim of obtaining the influence relationship between the increase in the shear heat and the change in the tank depth. We used COMSOL 5.6 software to simulate the movement of the screw during injection molding, obtained the temperature change of each section at the outer end of the screw, and compared the depth node temperature by reducing the depth of the base tank H by 10% to obtain the influence relationship between the increase in the shear heat, the change in screw groove depth and the aspect ratio.

Since the screw injection molding machine is in a continuous working state during injection production, its heating temperature, speed and other process parameters remain stable and unchanged, and a steady-state study is selected. Process control parameters specify the velocity and thermocouple heating temperature per unit time, and parametric sweep studies are added to the steady-state study to explore the influence of each process parameter on the process. Through the steady-state study mode under simulation, different arrangements and combinations of the process parameters such as the holding time, filling time, and clamping force are simulated to study their influence on the processing process. The quantitative reference indicators and results obtained are shown in Table 6, including but not limited to dependent variables such as the fluid flow rate, shear rate, temperature distribution and phase transition. Moreover, the shear heating process of the injection screw is considered in the mold flow analysis to ensure that the mold index value is more accurate. From the analysis results, it can be seen that the qualitative conclusion is that the actual degree of barrel offset is positively correlated with the temperature in the mold, the shear heat generation in the mold is positively correlated with the change in the groove depth of the screw, and the shear heat generation in the mold is positively correlated with the length-diameter ratio of the screw Thus, it can be inferred that with the increase in the groove depth and aspect ratio of the injection screw, the offset will also decrease (Supplmentary Information).

## Supplementary Information

Supplementary Information.

## Data Availability

All data generated or analysed during this study are included in this published article.

## References

[1] Chen, S.-C. *et al.* Study on the thermoforming of PC films used for in-mold decoration. *Int. Commun. Heat Mass Transf.* **35**(8), 967–973 (2008).

[2] Phillips, C. O., Claypole, T. C. & Gethin, D. T. Mechanical properties of polymer films used in in-mould decoration. *J. Mater. Process. Technol.* **200**(1–3), 221–231 (2008).

[3] Kim, G., Lee, K. & Kang, S. Prediction of the film thickness distribution and pattern change during film insert thermoforming. *Polym. Eng. Sci.* **49**(11), 2195–2203 (2009).

[4] Chen, S.-C. *et al.* Effect of decoration film on mold surface temperature during in-mold decoration injection molding process. *Int. Commun. Heat Mass Transf.* **37**(5), 501–505 (2010).

[5] Zhang, Y., Deng, Y. M. & Sun, B. S. Injection molding warpage optimization based on a mode-pursuing sampling method. *Polym. Plast. Technol. Eng.* **48**(7), 767–774 (2009).

[6] Martinez, A., Castany, J. & Aisa, J. Characterization of in-mold decoration process and influence of the fabric characteristics in this process. *Mater. Manuf. Process.* **26**(9), 1164–1172 (2011).

[7] Aguiar, R., Petel, O. E. & Miller, R. E. Effect of a Halloysite-polyurethane nanocomposite interlayer on the ballistic performance of laminate transparent armour. *Composites C* **7**, 1–10 (2022).

[8] Abtew, M. A. *et al.* Forming characteristics and surface damages of stitched multi-layered para-aramid fabrics with various stitching parameters for soft body armour design. *Compos. A Appl. Sci. Manuf.* **109**, 517–537 (2018).

[9] Mawkhlieng, U., Majumdar, A. & Laha, A. A review of fibrous materials for soft body armour applications. *RSC Adv.* **10**(2), 1066–1086 (2020).

[10] Crouch, I. G. Critical interfaces in body armour systems. *Defence Technol.* **17**(6), 1887–1894 (2021).

[11] Islam, M. K. *et al.* Biomimetic armour design strategies for additive manufacturing: A review. *Mater. Des.* **205**, 109750 (2021).

[12] Khare, S. *et al.* Determination of Johnson–Cook material parameters for armour plate using DIC and FEM. *Met. Mater. Int.* **27**(12), 4984–4995 (2020).

[13] Bajya, M. *et al.* Mitigating the blunt trauma of soft armour panels using polycarbonate sheets: A cost-effective solution. *Appl. Compos. Mater.* **28**(4), 1089–1109 (2021).

[14] Li, X. & Vaz, M. A. Analytical estimation on the number of bending cycles to initiate armour wires lateral buckling in flexible pipes. *Ocean Eng.* **228**, 108838 (2021).

[15] Bhat, A. *et al.* Advancement in fiber reinforced polymer, metal alloys and multi-layered armour systems for ballistic applications: A review. *J. Market. Res.* **15**, 1300–1317 (2021).

[16] Chang, H. *et al.* Non-dominant genetic algorithm for multi-objective optimization design of unmanned aerial vehicle shell process. *Polymers* **14**(14), 2896 (2022). 35890672 10.3390/polym14142896 PMC9322716

[17] Chang, H. *et al.* Using sequence-approximation optimization and radial-basis-function network for brake-pedal multi-target warping and cooling. *Polymers* **14**(13), 2578 (2022). 35808629 10.3390/polym14132578 PMC9269529

[18] Guo, W. *et al.* A combined in-mold decoration and microcellular injection molding method for preparing foamed products with improved surface appearance. *Polymers* **11**(5), 778 (2019). 31052446 10.3390/polym11050778 PMC6572461

